# Drug-Related Hospital Admissions *via* the Department of Emergency Medicine: A Cross-Sectional Study From the Czech Republic

**DOI:** 10.3389/fphar.2022.899151

**Published:** 2022-06-13

**Authors:** Zuzana Očovská, Martina Maříková, Jaromír Kočí, Jiří Vlček

**Affiliations:** ^1^ Department of Social and Clinical Pharmacy, Faculty of Pharmacy in Hradec Králové, Charles University, Hradec Králové, Czechia; ^2^ Department of Clinical Pharmacy, Hospital Pharmacy, University Hospital Hradec Králové, Hradec Králové, Czechia; ^3^ Department of Emergency Medicine, University Hospital Hradec Králové, Hradec Králové, Czechia

**Keywords:** adverse drug event, drug-related problem, hospitalization, prevalence, preventability, Czech Republic

## Abstract

**Background:** Drug-related hospital admissions (DRAs) represent a significant problem affecting all countries worldwide. This study aimed to determine the prevalence and preventability of DRAs, identify the most common medications involved in DRAs, the most common clinical manifestations of DRAs and describe the preventability aspects of DRAs.

**Methods:** This cross-sectional study examined unplanned hospital admissions to the University Hospital Hradec Králové *via* the department of emergency medicine in August–November 2018. Data were obtained from electronic medical records. The methodology of DRA identification was adapted from the OPERAM DRA adjudication guide.

**Results:** Out of 1252 hospital admissions, 195 DRAs have been identified (145 related to treatment safety, 50 related to treatment effectiveness). The prevalence of DRAs was 15.6% (95% CI 13.6–17.6). The most common medication classes involved in DRAs related to treatment safety were Antithrombotic agents, Antineoplastic agents, Diuretics, Corticosteroids for systemic use, and Beta blocking agents. The most common medication classes involved in DRAs related to treatment effectiveness included Diuretics, Antithrombotic agents, Drugs used in diabetes, Agents acting on the renin-angiotensin system, and Lipid modifying agents. Gastrointestinal disorders were the leading causes of DRAs related to treatment safety, while Cardiac disorders were the leading causes of DRAs related to treatment effectiveness. The potential preventability of DRAs was 51%. The highest share of potential preventability in medication classes repeatedly involved in DRAs related to treatment safety was observed for Anti-inflammatory and antirheumatic products, Psycholeptics, and Drugs used in diabetes. Potentially preventable DRAs related to treatment safety were most commonly associated with inappropriate drug selection, inappropriate monitoring, inappropriate dose selection, and inappropriate lifestyle measures. On the contrary, DRAs related to treatment effectiveness were more commonly associated with medication nonadherence.

**Conclusion:** It should be emphasized that in most DRAs, medications were only a contributory reason of hospital admissions and that benefits and risks have to be carefully balanced. It is highlighted by the finding that the same medication classes (Antithrombotic agents and Diuretics) were among the most common medication classes involved in DRAs related to treatment safety and simultaneously in DRAs related to treatment effectiveness. The study highlighted that apart from problems related to prescribing, problems related to monitoring and patient-related problems represent significant preventability aspects.

## Introduction

Drug-related hospital admissions (DRAs) represent a significant problem affecting all countries over the world. Although many studies have focused on adverse drug reactions (ADRs) leading to hospital admissions, fewer studies have addressed broader concepts, such as adverse drug events (ADEs) and drug-related problems (DRPs).

Multiple terms and definitions are used to describe medication harm in research and clinical practice (Falconer et al., 2019). ADEs could be defined as injuries caused by drug use that encompass ADRs and harm resulting from medication errors—they are the targets of broader efforts to improve patient safety (Nebeker et al., 2004).

A DRP is an event or circumstance involving drug therapy that actually or potentially interferes with desired health outcomes (Pharmaceutical Care Network Europe Association, 2020). DRPs are divided into two main domains—DRPs related to treatment effectiveness (problem with the effect of the pharmacotherapy) and DRPs related to treatment safety (patient suffers, or could suffer, from an ADE). The third domain (“Other”) includes unnecessary drug treatment (Pharmaceutical Care Network Europe Association, 2020).

While on the one hand, the use of medications might lead to ADEs, their use reduces hospital admissions as well. For example, the following medication classes were found to reduce emergency hospitalizations: angiotensin-converting enzyme inhibitors, angiotensin II receptor blockers, aldosterone receptor antagonists, statins, long-acting muscarinic antagonists, and long-acting beta-2 adrenoceptor agonists (Bobrovitz et al., 2018). Therefore, DRPs related to treatment effectiveness should also be the focus when studing DRAs.

So far, only a few studies have examined the extent to which DRPs contribute to hospital admissions. Recently, new tools (Thevelin et al., 2018; Kempen et al., 2019) have also incorporated DRPs related to treatment effectiveness. These include omission of an evidence-based drug, inappropriate selection of a drug or a dosage form, inappropriate administration, subtherapeutic dose, too short duration of treatment, medication nonadherence, inappropriate monitoring, inappropriate discontinuation, drug-drug interaction and drug-food interactions.

The concern should not only be minimizing the risks of pharmacotherapy, but also maximizing the effectiveness of pharmacotherapy (ensuring that the goals of treatment are reached). DRPs can be prevented primarily by appropriate pharmacotherapy (selection of medications and their formulation, dosing scheme, and duration of treatment—both prescribed and over-the-counter medications), appropriate use and administration of medications, appropriate medication adherence, appropriate monitoring (whether treatment goals are reached, risk factors of complications of the disease, occurrence of ADR and risk factors of ADRs), and appropriate lifestyle measures (e.g., fluid and food intake, smoking, alcohol consumption, sunscreen use).

As indicated by the definition of DRP, a DRP can be either potential (possibly leading to real problems for the patient) or actual/manifest (the problem already impacts the patient and his therapy) (Westerlund, 2019). Admission to the hospital can be a measurable outcome of manifest DRP.

Numerous studies have been conducted on DRAs from high-income countries. However, there are fewer studies from low- and middle-income countries and central and eastern Europe. This is the first study from the Czech Republic that examines DRAs without any department or age limit. In previous studies from the Czech Republic, the population studied was either from the pediatric ward (Langerová et al., 2014) or the geriatric ward (Maříková et al., 2021).

Reducing avoidable medication-related harm remains a difficult global patient safety challenge. Studies measuring the scope and nature of preventable ADEs can provide essential knowledge for the development of risk minimization measures.

The study aimed to provide information on:

a) the prevalence of DRAs to the University Hospital Hradec Králové *via* the department of emergency medicine,
b) the most common medications involved in DRAs,
c) the most common clinical manifestations of DRAs,
d) the potential preventability of DRAs,
e) medications most frequently associated with potentially preventable DRAs,
f) the most common clinical manifestations of potentially preventable DRAs, and
g) preventability aspects most frequently associated with potentially preventable DRAs.

## Methods

### Study Design and Setting

This observational cross-sectional study examined hospital admissions to the University Hospital Hradec Králové *via* the department of emergency medicine in order to identify those which are drug-related. Hospital admissions were identified using a register of all hospital admissions to the University Hospital Hradec Králové *via* the department of emergency medicine. Most of the patients were admitted to the departments of internal medicine (49%), surgery (26%), neurology (10%), pneumology (4%), anesthesiology, resuscitation and intensive medicine (3%), oncology and radiotherapy (3%), orthopedics (2%), infectious diseases (1%), and psychiatry (1%). The number of hospital admissions *via* the department of emergency medicine of the University Hospital Hradec Králové is approximately 450 per month.

The study followed the Strengthening the Reporting of Observational Studies in Epidemiology (STROBE) statement for the reporting of the study (von Elm et al., 2008).

### Inclusion and Exclusion Criteria

The study included all patients who were admitted *via* the department of emergency medicine to any hospital ward of University Hospital Hradec Králové. Hospital admissions that took place between 12th August and 6th November 2018 were included. Visits to the department of emergency medicine without inpatient hospitalization were not included. Hospitalizations for diagnostic or elective surgical procedures for pre-existing conditions, hospitalizations with missing medical records, and hospitalizations taking less than 24 h were excluded. There were no exclusion criteria related to age or department. Patients hospitalized more than once were counted as separate cases.

### Data Collection

The data collection process was retrospective. Data were obtained from electronic medical records and entered into a Microsoft Access database. The collected data included demographic characteristics, medication history, medical history, presenting complaint, admission diagnosis, laboratory values and results of clinical investigations, documented ADRs and information on medication adherence. Medications stated in medication history were counted as active substances.

### Ethics Committee Approval

The study was approved by Ethics Committee of the University Hospital Hradec Králové and Ethics Committee of the Faculty of Pharmacy in Hradec Králové. Patient informed consent was not required due to the observational design of the study and the retrospective data collection process. No personal data that could identify the patients were collected.

### Methods of Assessment

The methodology of DRA adjudication was adapted from the Drug-related admissions adjudication guide developed within the OPERAM project (Thevelin et al., 2018). The DRA identification process had the following steps: data abstraction, screening for potential ADEs causing or contributing to hospital admission, causality assessment, assessment of contribution to hospital admission, and the assessment of preventability.

Potential ADEs that caused or contributed to hospital admission were identified and the causality of each ADE was assessed using WHO-UMC criteria. The modified WHO-UMC causality criteria (Klopotowska et al., 2013) described in the Drug-related admissions adjudication guide (Thevelin et al., 2018) were used to assess causality due to underuse. In addition, dosage adjustments were taken into account. ADEs with certain causal relationships had to fulfill the following criteria: 1) plausible time relationship to drug intake/dose increase, 2) plausible response to withdrawal/dose decrease, 3) cannot be explained by any disease, 4) definitive pharmacologically or phenomenologically, and 5) satisfactory rechallenge. ADEs with probable causal relationship had to fulfill the following criteria: 1) reasonable time relationship to drug intake/dose increase, 2) clinically reasonable response to withdrawal/dose decrease, and 3) unlikely to be attributed to any disease. ADEs with possible causal relationships included events with a reasonable time relationship to drug intake/dose increase that could also be explained by disease or information on dechallenge was lacking or unclear. ADEs with certain, probable, or possible causal relationships were considered confirmed ADEs.

In case of a confirmed ADE, the ADE contribution to hospital admission was accessed. According to the definition of DRA, hospitalizations due to ADEs that were the main reason for admission, as well as ADEs that were a contributory reason for admission, were considered a DRA. The main reason for admission was the primary cause of admission and was usually documented in the admission or discharge letter. A contributory reason for admission was a clinically significant contributory factor to admission—an event that worsened the main reason for admission or played a substantial role in the admission, but other factors also contributed significantly to the admission.

Drug therapeutic failure without an evident cause, drug-related laboratory deviation without clinical manifestation, intentional intoxication, and ADE that was present at hospital admission but not related to the reason of admission were not considered a DRA.

The last step was the assessment of preventability. DRAs judged to be due to medication errors were deemed to be potentially preventable. Preventability was further assessed using Hallas criteria as definitely avoidable, possibly avoidable, not avoidable, and unevaluable (Hallas et al., 1990).

Preliminary screening for potential ADEs was performed by a PhD candidate in clinical pharmacy (ZO), and the consensus assessment was performed by three board-certified clinical pharmacists (MM, JV, PS).

### Classification

The identified DRAs were classified into two groups—DRAs related to treatment safety and DRAs related to treatment effectiveness. The Anatomical Therapeutic and Chemical (ATC) classification was used to code medications and medication groups (WHOCC, 2022). Medications were coded up to the fifth level. Medical Dictionary for Regulatory Activities (MedDRA) was used to classify clinical manifestations (BioPortal, 2021). MedDRA^®^ the Medical Dictionary for Regulatory Activities terminology is the international medical terminology developed under the auspices of the International Council for Harmonisation of Technical Requirements for Pharmaceuticals for Human Use (ICH).

Potentially preventable DRAs were classified according to the OPERAM DRA adjudication guide (Thevelin et al., 2018) into DRAs related to overuse, underuse, and misuse as well as the Pharmaceutical Care Network Europe Classification V 9.1 (Pharmaceutical Care Network Europe Association, 2020) into DRAs concerning the following DRPs: drug selection, dose selection, treatment duration, patient-related, patient transfer-related and other (No or inappropriate outcome monitoring). An additional category was added—inappropriate lifestyle measures.

### Outcome Measures

The main outcome measure was the prevalence of DRAs (defined as the number of unplanned DRAs divided by the total number of unplanned hospital admissions). DRA was defined as a hospitalization due to an ADE, which is the main or contributory reason for hospital admission of a patient. The term ADE was defined as harm due to an ADR or a medication error related to overuse, underuse, or misuse of prescription and non-prescription medications (Thevelin et al., 2018).

The other outcomes included: the prevalence of potentially preventable DRAs (defined as the number of potentially preventable DRAs divided by the total number of DRAs), the most common medication classes implicated in DRAs, the most common clinical manifestations of DRAs, the most common medication classes implicated in potentially preventable DRAs, the most common clinical manifestations of potentially preventable DRAs and preventability aspects of potentially preventable DRAs.

### Sample Size Calculation and Data Analysis

The following formula (Daniel and Cross, 2013) was used to calculate the sample size:

$$n=\frac{Z^{2}P\left(1-P\right)}{d^{2}}$$

where *p* stands for the expected prevalence, *Z* for the standard normal variable corresponding to the confidence interval (CI), and *d* for precision.

A sample size of 1252 patients was required to estimate the prevalence of DRAs, based on 95% CI, precision level of 2%, and the prevalence of 15.4% [obtained from the latest systematic review (Ayalew et al., 2019)].

Categorical variables were expressed as absolute values and percentages. Continuous variables were expressed as medians with interquartile ranges.

## Results

### Prevalence of Drug-Related Hospital Admissions and Sample Characteristics

**FIGURE 1:**
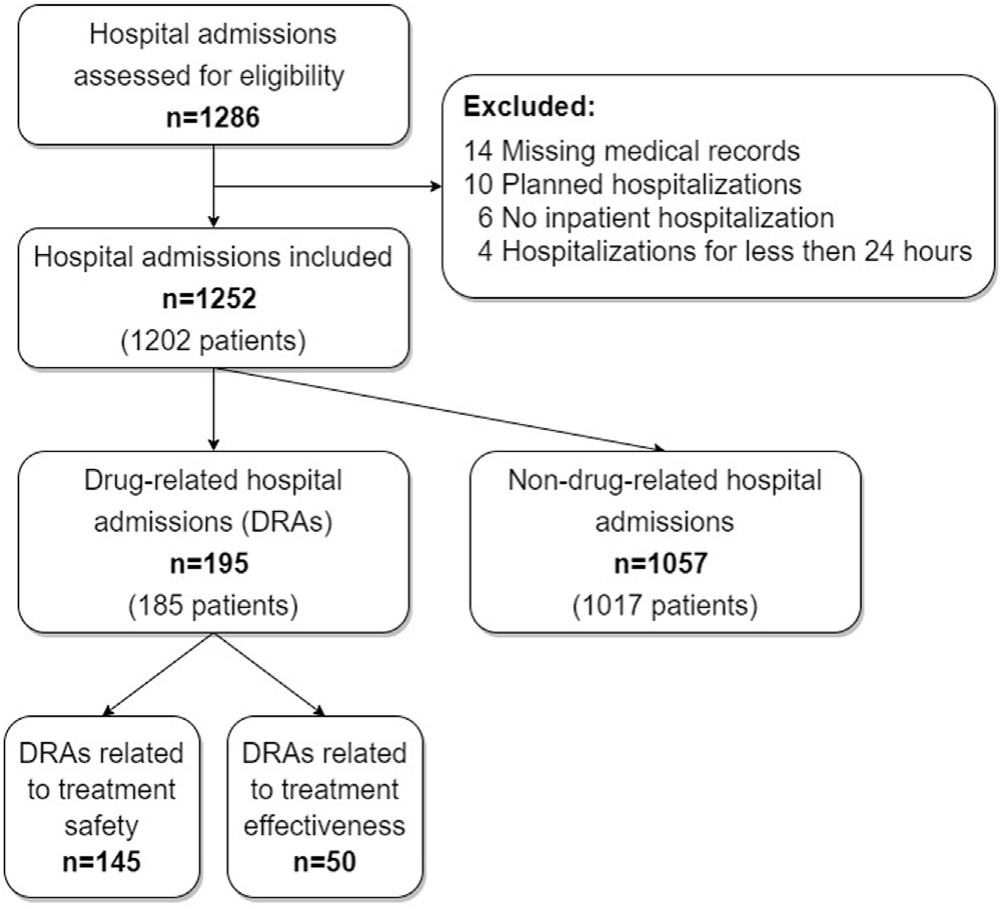
A flow diagram depicting the number of hospital admissions at each step of the study.

The study included 1252 unplanned hospital admissions to University Hospital Hradec Králové *via* the department of emergency medicine. The number of patients admitted to the hospital was 1202, as some patients were admitted more than once. A total of 195 hospital admissions were identified to be drug-related. Of the 195 DRAs, 145 DRAs (74%) were related to treatment safety, and 50 DRAs (26%) were related to treatment effectiveness. The total prevalence of DRAs was 15.6% (95% CI 13.6–17.6). For the flow diagram, see Figure 1.

The demographic and clinical characteristics of the study sample and the comparison of subgroups are shown in Table 1.

**TABLE 1:** Demographic and clinical characteristics of the study sample and the comparison of subgroups.

Characteristic	Total N = 1252	DRAs *n* = 195	DRAs related to		Non-DRAs *n* = 1057	DRAs related to safety	
			Treatment effectiveness *n* = 50	Treatment safety *n* = 145		Preventable *n* = 50	Non-preventable *n* = 95
Age							
Median	71	75	68	77	70	82	76
IQR	58–82	66–84	59–78	70–85	56–81	71–86	69–83
Sex							
Female—No. (%)	570 (46%)	91 (47%)	19 (38%)	72 (50%)	479 (46%)	27 (54%)	45 (47%)
Male—No. (%)	682 (54%)	104 (53%)	31 (62%)	73 (50%)	578 (55%)	23 (46%)	50 (53%)
Number of medications in medication history							
Median	5	8	5	9	5	8	10
IQR	2–9	5–11	3–8	6–12	1–8	5–10	7–13
Charlson comorbidity index							
Median	4	5	5	6	4	6	6
IQR	2–6	4–7	2–6	4–7	2–6	4–7	4–7
Estimated glomerular filtration rate							
Median	66	55	65	54	69	54	54
IQR	44–88	34–81	41–90	32–74	46–89	34–76	31–74
Body mass index							
Median	26	26	26	26	26	26	26
IQR	23–31	23–29	24–29	23–31	23–31	23–28	24–31

DRA, Drug-related hospital admission; IQR, interquartile range.

Table 2 shows the comorbidities of the study sample and the comparison of subgroups.

**TABLE 2:** Comorbidities of the study sample and inter-group differences.

Presence of comorbidity	Total *N* = 1252	DRAs *n* = 195	DRAs related to		Non-DRAs *n* = 1057	DRAs related to safety	
			Treatment effectiveness *n* = 50	Treatment safety *n* = 145		Preventable *n* = 50	Non-preventable *n* = 95
Arterial hypertension	60%	75%	70%	77%	57%	74%	79%
Dyslipidemia	34%	41%	28%	45%	33%	40%	47%
Diabetes	28%	38%	40%	37%	26%	34%	39%
Coronary artery disease	21%	29%	32%	28%	20%	28%	28%
Valvular heart disease	19%	33%	38%	32%	16%	22%	37%
Atrial fibrillation	17%	31%	22%	34%	15%	30%	37%
Vertebrogenic algic syndrome	17%	27%	14%	31%	16%	36%	28%
Tumors	17%	23%	6%	28%	16%	20%	33%
Heart failure	14%	26%	32%	24%	12%	24%	24%
Chronic kidney disease	13%	24%	10%	29%	11%	30%	28%
Hyperuricemia/gout	11%	13%	4%	16%	11%	16%	16%
Osteoarthrosis	11%	12%	8%	14%	11%	16%	13%
Benign prostatic hyperplasia	11%	16%	16%	16%	10%	18%	15%
Hypothyreosis	10%	13%	10%	14%	9%	16%	14%
Anemia	9%	17%	10%	19%	8%	16%	21%
Chronic venous insufficiency	9%	16%	16%	17%	8%	20%	15%
Dementia	9%	10%	6%	12%	8%	16%	9%
Venous thromboembolism	8%	13%	16%	12%	7%	16%	11%
Depression/anxiety	8%	11%	18%	9%	7%	10%	8%
Liver disease	8%	13%	18%	12%	7%	12%	12%
Peripheral artery disease	7%	11%	8%	12%	7%	14%	12%
Chronic obstructive pulmonary disease	7%	10%	14%	8%	7%	2%	12%
Osteoporosis	7%	10%	2%	13%	6%	10%	15%
Peptic ulcer	6%	9%	4%	11%	6%	20%	6%
Heart arrhythmia	6%	6%	6%	6%	6%	6%	5%
Gastroesophageal reflux disease	6%	8%	6%	9%	6%	8%	9%
Asthma	6%	6%	8%	5%	6%	4%	5%
Obesity	27%	22%	16%	24%	28%	18%	27%
Overweight	31%	35%	42%	33%	31%	32%	34%
Tobacco smoking	17%	15%	28%	11%	17%	16%	8%
Alcohol consumption	10%	11%	26%	6%	10%	8%	5%
Immobility	5%	7%	2%	9%	4%	12%	7%

DRA: Drug-related hospital admission.

Note: Comorbidities with <2% prevalence were omitted from this table for readability.

Table 3 shows the number of hospital admissions with corresponding medication classes in the patients’ medication history and the comparison of subgroups.

**TABLE 3:** Baseline medications grouped by ATC group and inter-group differences.

ATC group	Total *N* = 1252	DRAs *n* = 195	DRAs related to		non-DRAs *n* = 1057	DRAs related to safety	
			Treatment effectiveness *n* = 50	Treatment safety *n* = 145		Preventable *n* = 50	Non-preventable *n* = 95
Diuretics	58%	90%	92%	89%	52%	70%	99%
Antithrombotic agents	53%	78%	52%	88%	48%	84%	89%
Agents acting on the renin-angiotensin system	40%	43%	40%	44%	40%	44%	44%
Drugs used in diabetes	36%	51%	60%	48%	33%	46%	48%
Beta blocking agents	35%	44%	34%	48%	33%	42%	51%
Drugs for obstructive airway diseases	32%	32%	30%	32%	33%	16%	41%
Lipid modifying agents	32%	35%	28%	37%	32%	34%	39%
Drugs for acid related disorders	30%	46%	26%	52%	27%	48%	55%
Analgesics	23%	39%	8%	50%	20%	50%	51%
Calcium channel blockers	22%	25%	16%	28%	22%	32%	25%
Psychoanaleptics	22%	27%	28%	26%	21%	24%	27%
Psycholeptics	21%	27%	16%	30%	20%	44%	23%
Mineral supplements	16%	24%	10%	29%	15%	22%	33%
Antigout preparations	15%	19%	6%	24%	14%	20%	26%
Cardiac therapy	13%	26%	16%	29%	11%	16%	36%
Urologicals	13%	19%	14%	21%	11%	16%	23%
Thyroid therapy	12%	14%	4%	17%	12%	18%	17%
Vitamins	12%	18%	14%	19%	11%	12%	23%
Antiepileptics	8%	14%	6%	17%	7%	18%	16%
Vasoprotectives	7%	10%	6%	11%	7%	16%	8%
Anti-inflammatory and antirheumatic products	7%	14%	10%	16%	6%	32%	7%
Antihypertensives	6%	8%	4%	10%	5%	14%	7%
Antianemic preparations	6%	9%	10%	8%	5%	8%	8%
Drugs for functional gastrointestinal disorders	6%	11%	6%	12%	5%	10%	14%
Corticosteroids for systemic use	5%	13%	4%	16%	4%	4%	22%
Antineoplastic agents	5%	18%	0%	25%	2%	4%	36%
Antihistamines for systemic use	5%	8%	4%	9%	4%	4%	12%
Ophthalmologicals	5%	6%	0%	8%	4%	8%	7%
Laxatives	3%	4%	0%	6%	3%	6%	5%
Immunosuppressants	3%	7%	4%	8%	2%	2%	12%
Cough and cold preparations	3%	4%	0%	5%	2%	0%	7%
Anti-parkinson drugs	2%	5%	4%	6%	2%	6%	5%
Other nervous system drugs	2%	0%	0%	0%	3%	0%	0%
Antidiarrheals, intestinal antiinflammatory/antiinfective agents	2%	6%	6%	6%	2%	4%	6%
Drugs for treatment of bone diseases	2%	2%	0%	2%	2%	4%	1%

DRA, Drug-related hospital admission; ATC: Anatomical Therapeutic Chemical.

Note: Medication classes with <2% prevalence were omitted from this table for readability.

### Clinical Manifestation of Drug-Related Hospital Admissions

A total of 152 ADEs were related to treatment safety. More than one ADE was identified in 7 DRAs. Table 4 shows the MedDRA classification of ADEs related to treatment safety.

**TABLE 4:** MedDRA classification of ADEs related to treatment safety (N = 152).

MedDRA system organ class (No., %)	MedDRA preferred term	No.
Gastrointestinal disorders (31, 20.4%)	Gastroduodenal hemorrhage	10
	Intestinal hemorrhage	7
	Diarrhea	3
	Gastric ulcer perforation	3
	Pancreatitis	1
	Gastrooesophageal reflux disease	1
	Gastritis	1
	Esophagitis	1
	Duodenal perforation	1
	Abdominal discomfort	1
	Dyspepsia	1
	Nausea	1
Metabolism and nutrition disorders (26, 17.1%)	Hyponatremia	12
	Hypoglycemia	6
	Hyperglycemia	3
	Dehydration	2
	Hyperkalemia	2
	Calciphylaxis	1
Blood and lymphatic system disorders (18, 11.8%)	Bone marrow toxicity	10
	Microcytic anemia	7
	Anemia folate deficiency	1
Nervous system disorders (17, 11.1%)	Cerebral hemorrhage	6
	Depressed level of consciousness	8
	Subdural hemorrhage	2
	Diplopia	1
Infections and infestations (14, 9.2%)	Infection susceptibility increased	10
	*Clostridium difficile* colitis	4
Cardiac disorders (12, 7.9%)	Bradycardia	7
	Atrioventricular block	3
	Hypertension	1
	Cardiomyopathy	1
Vascular disorders (10, 6.6%)	Hypotension	4
	Hematoma	3
	Syncope	2
	Hemorrhage	1
Renal and urinary disorders (8, 5.3%)	Hematuria	4
	Prerenal failure	4
Respiratory, thoracic, and mediastinal disorders (7, 4.6%)	Hemoptysis	3
	Pulmonary embolism	1
	Pulmonary alveolar hemorrhage	1
	Interstitial lung disease	1
	Epistaxis	1
Immune system disorders (4, 2.6%)	Drug hypersensitivity	4
Psychiatric disorders (2, 1.3%)	Confusional state	1
	Disorientation	1
Endocrine disorders (1, 0.7%)	Sec. adrenocortical insufficiency	1
General disorders and administration site conditions (1, 0.7%)	Fatigue	1
Injury, poisoning, and procedural complications (1, 0.7%)	Fall	1

ADE, Adverse Drug Event; MedDRA, Medical Dictionary for Regulatory Activities.

Table 5 shows the classification of DRAs related to treatment effectiveness according to MedDRA.

**TABLE 5:** MedDRA classification of DRAs related to treatment effectiveness (N = 50).

MedDRA system organ class	No.	%	MedDRA preferred term	No.
Cardiac disorders	16	32	Heart failure signs and symptoms	14
			Myocardial infarction	2
Nervous system disorders	9	18	Ischemic stroke	9
Metabolism and nutrition disorders	9	18	Diabetic complication	9
Vascular disorders	6	12	Venous thrombosis	3
			Hypertension	2
			Granulomatosis with polyangiitis	1
Blood and lymphatic system disorders	3	6	Anemia	3
Infections and infestations	2	4	Infection	2
Immune system disorders	2	4	Crohn’s disease	2
Psychiatric disorders	2	4	Depression	1
			Bipolar disorder	1
Endocrine disorders	1	2	Thyrotoxic crisis	1

DRA, Drug-related hospital admission; MedDRA, Medical Dictionary for Regulatory Activities.

### Medications Involved in Drug-Related Hospital Admissions Related to Treatment Safety

Table 6 shows the ATC classification of medication classes involved in DRAs related to treatment safety. A total of 254 medications were involved in ADEs related to treatment safety. The medications classes most frequently concerned the Cardiovascular system (27%), Blood and blood forming organs (26%), Antineoplastic and immunomodulating agents (16%), and Nervous system (11%). More than one medication was involved in 70 (48%) DRAs related to treatment safety.

**TABLE 6:** ATC classification of medication classes involved in DRAs related to treatment safety (N = 254).

ATC code	ATC group	No.	%
B01	Antithrombotic agents	65	25.6
L01	Antineoplastic agents	30	11.8
C03	Diuretics	28	11.0
H02	Corticosteroids for systemic use	14	5.5
C07	Beta blocking agents	14	5.5
M01	Anti-inflammatory and antirheumatic products	13	5.1
C09	Agents acting on the renin-angiotensin system	13	5.1
N02	Analgesics	11	4.3
L04	Immunosuppressants	10	3.9
J01	Antibacterials for systemic use	9	3.5
A10	Drugs used in diabetes	8	3.1
C01	Cardiac therapy	8	3.1
N03	Antiepileptics	5	2.0
N06	Psychoanaleptics	5	2.0
N05	Psycholeptics	5	2.0
C08	Calcium channel blockers	3	1.2
R03	Drugs for obstructive airway diseases	2	0.8
C02	Antihypertensives	2	0.8
M03	Muscle relaxants	2	0.8
A12	Mineral supplements	1	0.4
A07	Antidiarrheals, intestinal anti-inflammatory/antiinfective agents	1	0.4
H01	Pituitary and hypothalamic hormones and analogues	1	0.4
R05	Cough and cold preparations	1	0.4
N04	Anti-parkinson drugs	1	0.4
G03	Sex hormones and modulators of the genital system	1	0.4
G04	Urologicals	1	0.4

DRA, Drug-related hospital admission; ATC, Anatomical Therapeutic Chemical.

The most common medications involved in DRAs related to treatment safety included low dose acetylsalicylic acid (n = 23), warfarin (n = 22), prednisone (n = 8), hydrochlorothiazide (n = 8), clopidogrel (n = 7), furosemide (n = 7), perindopril (n = 6), insulin (n = 6), amiodarone (n = 5), bisoprolol (n = 5), ibuprofen (n = 5), nadroparin (n = 5), and spironolactone (n = 5).

### Medications Involved in Drug-Related Hospital Admissions Related to Treatment Effectiveness

Table 7 shows the ATC classification of the medication classes involved in 50 DRAs related to treatment effectiveness (N = 62). There were 9 DRAs related to treatment effectiveness in which more than one medication class was involved.

**TABLE 7:** ATC classification of medication classes involved in DRAs related to treatment effectiveness (N = 62).

ATC code	ATC group	No.	%
C03	Diuretics	14	22.6
B01	Antithrombotic agents	12	19.4
A10	Drugs used in diabetes	8	12.9
C09	Agents acting on the renin-angiotensin system	7	11.3
C10	Lipid modifying agents	5	8.1
C07	Beta blocking agents	3	4.8
J01	Antibacterials for systemic use	3	4.8
B03	Antianemic preparations	3	4.8
L04	Immunosuppressants	2	3.2
C08	Calcium channel blockers	1	1.6
A07	Intestinal antiinflammatory agents	1	1.6
H03	Thyroid therapy	1	1.6
N06	Psychoanaleptics	1	1.6
N05	Psycholeptics	1	1.6

DRA, Drug-related hospital admission; ATC, Anatomical Therapeutic Chemical.

### Causality Assessment

Causality was assessed for every event separately. There were 7 DRAs, with more than 2 ADEs contributing to hospital admission. According to the causality assessment, 51% ADEs were probable, and 49% ADEs were possible. No ADE was certain, as no event was a recognized pharmacological phenomenon, and rechallenge was almost never performed. ADEs with probable causality were events unlikely to be attributed to disease, and the response to withdrawal (or drug initiation) was clinically reasonable, while ADEs with possible causality included events that could also be explained by disease or the information on withdrawal (or drug initiation) was lacking or unclear. Table 8 shows the categories of causal relationships of ADEs involved in DRAs. Within DRAs related to treatment effectiveness, 46% of events had a probable causal relationship. Within DRAs related to treatment safety, 53% of events had a probable causal relationship.

**TABLE 8:** Causality assessment of ADEs.

Causality category	All ADEs *N* = 202	Treatment safety *n* = 152	Treatment effectiveness *n* = 50
probable	104	81	23
possible	98	71	27

ADE, Adverse drug events.

### Contribution to Hospital Admissions

In 55% of DRAs, ADEs only contributed to the admission, which means that ADE was one factor among others that together resulted in hospitalization. The most common other factors were heart failure decompensation and infection. Table 9 shows the categories of contributions to hospital admissions.

**TABLE 9:** Classification of DRAs—contribution to hospital admissions.

Contribution to hospital admission	All DRAs *N* = 195	Treatment safety *n* = 145	Treatment effectiveness *n* = 50
main reason	88	55	33
contributory reason	107	90	17

DRA, Drug-related hospital admission.

### Potentially Preventable Drug-Related Hospital Admissions

The overall potential preventability of DRAs was 51.3% (both definitely avoidable and possibly avoidable DRAs). We have identified 50 potentially preventable DRAs related to treatment safety and 50 potentially preventable DRAs related to treatment effectiveness. In addition, 83 (43%) DRAs were not avoidable, and 12 (6%) DRAs were unevaluable.

Table 10 shows the classification of preventable DRAs related to treatment safety. Regarding treatment safety, the most common preventability aspects included inappropriate drug selection, inappropriate monitoring, inappropriate dose selection, and inappropriate lifestyle measures.

**TABLE 10:** Classification of potentially preventable DRAs related to treatment safety (N = 50).

Categories of preventable DRAs related to treatment safety	No.	Medication involved
OVERUSE		
Drug without an indication	4	low-dose acetylsalicylic acid (3), levodopa
UNDERUSE		
Omission of an indicated drug	3	omission of gastric acid suppressants despite prior gastritis or gastrointestinal ulcer naproxen, ibuprofen, ibuprofen (+rivaroxaban)
MISUSE		
Wrong drug	13	nimesulide, furosemide
• inappropriate according to guidelines		
• contraindication or precaution for a certain condition with increased risk of toxicity		diclofenac (2), meloxicam (2), ibuprofen (2), nimesulide, amiodarone, bisoprolol, dosulepin, doxazosin
Wrong dose	9	glimepiride, tramadol
• the dose was too high		
• the dose was not adapted to the patient characteristics (age, renal function, weight)		diclofenac, tiapride, nadroparin
• the dose was given too frequently		metoprolol
• accidentally ingesting a toxic amount of drug		tramadol + zolpidem, tiapride, insulin
Inappropriate monitoring	11	amiodarone (+bisoprolol), verapamil, digoxin (+nebivolol)
• symptoms of bradycardia, heart rate		
• symptoms of bleeding, INR		warfarin (5)
• blood glucose		insulin (2)
• blood potassium		potassium chloride
Drug-drug interactions	1	haloperidol (+morphine, fentanyl)
OTHER		
Inappropriate lifestyle measures	9	glimepiride, insulin
• food intake		
• fluid intake		furosemide (2), digoxin, amiloride (+telmisartan), perindopril
• smoking		hormonal contraceptives
• heavy episodic alcohol consumption		warfarin

DRA, Drug-related hospital admission; INR, International Normalized Ratio.

Table 11 shows the classification of preventable DRAs related to treatment effectiveness. The most common preventability aspect of DRAs related treatment effectiveness was medication nonadherence.

**TABLE 11:** Classification of potentially preventable DRAs related to treatment effectiveness (N = 50).

Categories of preventable DRAs related to treatment effectiveness	No.	Medication classes involved
UNDERUSE		
Omission of the indicated drug	8	Antithrombotic agents (2), Antithrombotic agents + Lipid modifying agents (2), Agents acting on the renin-angiotensin system (1), Antianemic preparations (1), Thyroid therapy (1), Diuretics + Agents acting on the renin-angiotensin system (1)
The duration of therapy is too short	1	Antithrombotic agents (1)
Adherence concerns	35	Diuretics (7), Drugs used in diabetes (6), Agents acting on the renin-angiotensin system (4), Antibacterials for systemic use (3), Antithrombotic agents (3), Antianemic preparations (2), Immunosuppressants (2), Antithrombotic agents + Lipid modifying agents (1), Calcium channel blockers + Antithrombotic agents (1), Calcium channel blockers + Beta blocking agents + Diuretics + Lipid modifying agents (1), Diuretics + Agents acting on the renin-angiotensin system + Antithrombotic agents + Lipid modifying agents (1), Diuretics + Beta blocking agents (1), Intestinal antiinflammatory agents (1), Psychoanaleptics (1), Psycholeptics (1)
MISUSE		
Inappropriate monitoring	5	Drugs used in diabetes (2), Diuretics (2), Antithrombotic agents (1)
Inappropriate discontinuation or dose decrease	1	Diuretics + Beta blocking agents (1)

DRA, Drug-related hospital admission.

Potentially preventable DRAs were also classified according to the Pharmaceutical Care Network Europe classification of DRPs (Supplementary Tables S1, S2).

### Medications Involved in Preventable Drug-Related Hospital Admissions

Medications associated with potentially preventable DRAs related to treatment safety are listed in Table 12.

**TABLE 12:** Medication classes involved in potentially preventable DRAs related to treatment safety (N = 51).

Medication classes	No.	Medications
Anti-inflammatory and antirheumatic products	12	ibuprofen (4), diclofenac (3), meloxicam (2), nimesulide (2), naproxen (1)
Antithrombotic agents	10	warfarin (6), acetylsalicylic acid (3), nadroparin (1)
Drugs used in diabetes	6	insulin (4), glimepiride (2)
Cardiac therapy	4	digoxin (2), amiodarone (2)
Diuretics	4	furosemide (3), amiloride (1)
Psycholeptics	4	tiapride (2), haloperidol (1), zolpidem (1)
Analgesics	2	tramadol (2)
Beta blocking agents	2	metoprolol (1), bisoprolol (1)
Agents acting on the renin-angiotensin system	1	perindopril
Antihypertensives	1	doxazosin
Anti-parkinson drugs	1	levodopa
Calcium channel blockers	1	verapamil
Mineral supplements	1	potassium chloride
Psychoanaleptics	1	dosulepin
Sex hormones and modulators of the genital system	1	hormonal contraceptive

DRA, Drug-related hospital admission.

The highest share of potential preventability in medication classes repeatedly involved in DRAs related to treatment safety was observed for Anti-inflammatory and antirheumatic products, Psycholeptics, and Drugs used in diabetes. For detailed information, see Table 13.

**TABLE 13:** Medication classes and corresponding share of preventability of DRAs related to treatment safety.

Medication classes repeatedly involved in DRAs related to treatment safety	DRAs related to treatment safety (No.)	Preventable DRAs related to treatment safety (No.)	Share (%)
Anti-inflammatory and antirheumatic products	13	12	92
Psycholeptics	5	4	80
Drugs used in diabetes	8	6	75
Antihypertensives	2	1	50
Cardiac therapy	8	4	50
Calcium channel blockers	3	1	33
Psychoanaleptics	5	1	20
Analgesics	11	2	18
Antithrombotic agents	65	10	15
Diuretics	28	4	14
Beta blocking agents	14	2	14
Agents acting on the renin-angiotensin system	13	1	8

DRA, Drug-related hospital admission.

Note: Medication classes involved only once in DRAs and medication classes that were not involved in preventable DRAs related to treatment safety were excluded.

### Medications Involved in Non-preventable Drug-Related Hospital Admissions

The most common medication classes involved in non-preventable DRAs included Antithrombotic agents (24%), Antineoplastic agents (19%), Diuretics (11%), Corticosteroids for systemic use (8%), Immunosuppressants (6%), Antibacterials for systemic use (5%), Beta blocking agents (5%), and Agents acting on the renin-angiotensin system (5%).

### Clinical Manifestations Associated With Potentially Preventable Drug-Related Hospital Admissions

The most common clinical manifestations associated with potentially preventable DRAs related to treatment safety were Hypoglycemia (6), Gastroduodenal hemorrhage (6), Depressed level of consciousness (5), and Bradycardia (4). The MedDRA classification is shown in Table 14.

**TABLE 14:** MedDRA categories of preventable DRAs related to treatment safety (N = 50).

MedDRA system organ class	No.	%	MedDRA preferred term
Gastrointestinal disorders	14	28	Gastroduodenal hemorrhage (6), Gastric ulcer perforation (2), Intestinal hemorrhage (2), Esophagitis (1), Diarrhea (1), Gastritis (1), Nausea (1)
Metabolism and nutrition disorders	10	20	Hypoglycemia (6), Hyperkalemia (2), Dehydration (2)
Nervous system disorders	9	18	Cerebral hemorrhage (2), Subdural hemorrhage (2), Depressed level of consciousness (5)
Cardiac disorders	5	10	Bradycardia (4), Atrioventricular block (1)
Vascular disorders	3	6	Hematoma (2), Syncope (1)
Respiratory, thoracic, and mediastinal disorders	3	6	Pulmonary embolism (1), Pulmonary alveolar hemorrhage (1), Hemoptysis (1)
Renal and urinary disorders	2	4	Prerenal failure (2)
Blood and lymphatic system disorders	2	4	Microcytic anemia (2)
Psychiatric disorders	1	2	Disorientation (1)
General disorders and administration site conditions	1	2	Fatigue (1)

MedDRA, Medical Dictionary for Regulatory Activities; DRA, Drug-related hospital admission.

## Discussion

The aims of the study (prevalence of DRAs, medications involved in DRAs, clinical manifestations of DRAs, preventability of DRAs, and preventability aspects) are discussed separately:

### Prevalence of Drug-Related Hospital Admissions

Epidemiological studies demonstrate that the burden of ADRs in both inpatient and outpatient settings is substantial (Bouvy et al., 2015). As the population is aging and multimorbidity and polypharmacy are increasing, one would expect the prevalence of DRAs to rise as well. However, at the same time, safer alternatives are being used in clinical practice, high-risk medications are being withdrawn from the market, and preventative measures are being implemented in clinical practice. The prevalence of DRAs differs due to inconsistencies in the definitions and methods of DRA identification (Leendertse et al., 2010; Linkens et al., 2020; Laatikainen et al., 2021), the selected threshold of causality assessment (Wallerstedt et al., 2021), patient population (Beijer and de Blaey, 2002; Leendertse et al., 2010; Laatikainen et al., 2021) and whether the denominator includes all admissions, only acute admissions, or specific wards (Leendertse et al., 2010). When comparing the prevalence of DRAs, one has to take all these things into account. Due to the current heterogeneity, it is practically impossible to compare the prevalences of DRAs among different studies. We found that 15.6% of acute hospital admissions were drug-related. The prevalence of DRAs related to treatment safety was found to be 11.6%. If we excluded the cases with possible causality, the prevalence would be 6%. If we limited the finding only to ADEs with a probable causal relationship which was the main reason for hospital admission related to treatment safety, the prevalence would be 3%. The results of the subgroup analysis can be found in Supplementary Table S3. A noteworthy difference is between different age groups. Among older patients (65 years or older), the prevalence of DRAs was 18.6% while the prevalence of DRAs among the rest of the patients was 10%. The prevalence of DRAs among patients aged 75 years or older was 20%.

This study followed the OPERAM DRA adjudication guide (Thevelin et al., 2018), which was interested in DRPs that cause harm. To differentiate between potential DRPs and manifest DRPs, the term ADEs was used for manifest DRPs. However, the term was also applied to DRP related to treatment effectiveness. One could argue that manifest DRPs related to treatment effectiveness should not be called ADEs, since ADE is mostly defined as an injury resulting from the use of a drug, and the term ADE does not include failure to use a drug (Nebeker et al., 2004). Another confusion comes when comparing ADRs and ADEs. Some studies use the definition of ADR as a noxious and unintended response to a drug, which occurs at doses normally used, while others drop the part about normally used doses or use other definitions. Therefore, one must be cautious even when comparing studies with the same outcomes, as they might be using different definitions. There is a pressing need for further discussion and international consensus on this topic (Falconer et al., 2019).

### Medications Implicated in Drug-Related Hospital Admissions

Several studies have revealed that DRAs are caused by commonly used medications. In our study, the most common medication classes involved in DRAs related to treatment safety were Antithrombotic agents, Antineoplastic agents, Diuretics, Corticosteroids for systemic use, Beta blocking agents, Anti-inflammatory and antirheumatic products, and Agents acting on the renin-angiotensin system. The OPERAM trial has found Diuretics and Antithrombotic agents to be the most frequently involved or omitted medication classes in DRAs (Blum et al., 2021). Summarizing our findings on DRAs related to treatment effectiveness and DRAs related to safety, we have found the same medication classes (Antithrombotic agents and Diuretics) to be most frequently involved in DRAs.

Regarding preventable DRAs related to treatment safety, the most common medication classes identified in our study were Anti-inflammatory and antirheumatic products, Antithrombotic agents, Drugs used in diabetes, Diuretics, Cardiac therapy, Psycholeptics, Analgesics, and Beta blocking agents. Similar findings were reported in a systematic review (Howard et al., 2007), which identified antiplatelets, diuretics, non-steroidal anti-inflammatory drugs (NSAIDs), anticoagulants, opioid analgesics, drugs affecting the renin-angiotensin system, and beta-blockers as the medication classes most commonly involved in preventable DRAs related to ADRs and overtreatment.

Regarding preventable DRAs related to treatment effectiveness, the systematic review by Howard et al. identified diuretics, antiepileptics, drugs used in diabetes, and beta-blockers to be most commonly involved in DRAs. A systematic review of prospective observational studies (Mongkhon et al., 2018) identified medications targeting the cardiovascular system, respiratory system, central nervous system, endocrine system, and medication used to treat infections to be most commonly associated with hospital admissions due to medication nonadherence. In our study, the most common medication classes were Diuretics, Antithrombotic agents, Drugs used in diabetes, and Agents acting on the renin-angiotensin system.

#### Comparison With Other Countries

Compared to lower-income countries, we have observed a lower prevalence of DRAs related to Antiinfectives for systemic use. Antiinfectives for systemic use were frequently involved in DRAs in Ethiopia (Angamo et al., 2017; Demessie and Berha, 2022), South Africa (Mouton et al., 2016), Nigeria (Adedapo et al., 2021), and India (Geer et al., 2016). Antiinfectives for systemic use were also frequently implicated in DRAs in Brazil (de Paula et al., 2012) during the time when the requirement to be prescription only was not met. In higher-income countries, Antiinfectives for systemic use are among the top medication classes among the pediatric population. A review comparing ADR-related hospitalizations in developed and developing countries (Angamo et al., 2016) found that antiinfectives were more commonly reported to be associated with ADR-related admissions in developing countries than in developed countries.

Compared to certain higher-income countries, Opioids were not among the most common medication classes involved in DRAs related to treatment safety. Opioids appear to be frequently involved in the United States (Budnitz et al., 2011; Poudel et al., 2017), Australia (Zhang et al., 2019), Canada (Bayoumi et al., 2014). A possible explanation could be that strong opioids are not yet widely prescribed in the Czech Republic compared to these countries. However, hospital admissions due to tramadol were also present in our setting. Otherwise, the same medication classes continue to be involved in DRAs in different countries.

### Clinical Manifestations of Drug-Related Hospital Admissions

Clinical manifestations of DRAs related to treatment safety most frequently concerned Gastrointestinal disorders (especially Gastrointestinal hemorrhage), Metabolism and nutrition disorders (especially Hyponatremia, Hypoglycemia) and Blood and lymphatic system disorders (Bone marrow toxicity, Microcytic anemia), Nervous system disorders (Depressed level of consciousness), Infections and infestations (Increased infection susceptibility) and Cardiac disorders (Bradycardia). Gastrointestinal disorders and Microcytic anemia were associated with anticoagulants, antiplatelets, and NSAIDs. Hyponatremia was associated with the use of thiazide diuretics. Hypoglycemia was associated with the use of insulin and sulfonylureas. Bone marrow toxicity was associated with the use of antineoplastic agents. A depressed level of consciousness was associated with opioid analgetics. Increased susceptibility to infection was associated with immunosuppressants. Bradycardia was associated with beta-blockers, amiodarone, and digoxin.

Clinical manifestation of DRAs related to treatment effectiveness most frequently concerned Cardiac disorders (particularly Heart failure symptoms), followed by Nervous system disorders (Ischemic stroke) and Metabolism and nutrition disorders (Diabetic complications). Heart failure symptoms were associated with the underuse of diuretics. Ischemic stroke due to cardioembolism was associated with the underuse of anticoagulants, while ischemic stroke due to atherosclerosis was associated with the underuse of antiplatelet agents, statins, and antihypertensive therapy. Diabetic complications were associated with nonadherence to antidiabetics.

Similarly, a study in the United Kingdom (Rogers et al., 2009) identified heart failure and stroke to be the most frequent manifestations of DRAs due to undertreatment. In a study in Belgium (Somers et al., 2010), the most common symptom associated with drug therapy failures was dyspnea. A study from Australia (Kalisch Ellett et al., 2021) identified that chronic heart failure and osteoporosis were most frequently associated with potentially suboptimal medication-related processes of care related to the underuse of medications. However, there are not many studies that focus not only on DRAs related to treatment safety but also on DRAs related to treatment effectiveness.

### Preventability of Drug-Related Hospital Admissions

We have found that half of DRAs were potentially preventable. However, in the subgroup of DRAs related to treatment safety, only 34% of DRAs were found to be preventable. Meta-analysis on the preventability of ADRs (Hakkarainen et al., 2012) found that half of ADRs among adult outpatients can be prevented.

Recent studies have also observed higher preventability: 60.9% (Li et al., 2021) 42.9% (Dechanont et al., 2021), 53.5% (Maříková et al., 2021), 46% (Kalisch Ellett et al., 2021) 47% (Lombardi et al., 2020), 76.4% (Cabre et al., 2018) 69% (Giardina et al., 2018). However, most of them were limited to older patients, in whom the preventability is higher than in the general population.

Like the prevalence of DRAs, the prevalence of preventable DRAs varies according to many factors. The inclusion of indirect drug-related causes for patient morbidity (errors of omission) and average sample age is associated with a higher prevalence of preventable DRAs (Winterstein et al., 2002). Variations can also be explained by differences in study populations and data collection methods (Patel et al., 2017).

### Preventability Aspects

A systematic review (Howard et al., 2007) identified problems with patient adherence to medication (33.3%) and prescribing problems (30.6%) as the most common underlying causes of preventable DRAs, followed by monitoring problems (22.2%).

Taking the results of DRAs related to treatment safety and treatment effectiveness together, our study confirms these findings. In our study, 38% of preventable DRAs concerned medication adherence problems, 35% concerned prescribing problems (drug selection, dosage selection, treatment duration) 17% inappropriate monitoring. Furthermore, 1% were related to medication reconciliation problems and 9% were related to inappropriate lifestyle measures (fluid intake, food intake, alcohol consumption, and smoking).

Similar underlying causes were also observed in a recent study on medication-related hospital readmissions (Uitvlugt et al., 2021), which found that 35% of preventable readmissions were due to prescribing errors, and 35% of preventable readmissions were due to nonadherence. Uitvlught et al. had pointed out that if patients present at the emergency department due to nonadherence, this will typically manifest itself as a worsening of their underlying disease, and only if the patient indicates that they are not adherent, this will be recognized as an ADE. Additionally, Uitvlugt et al. had found that 30% of preventable readmissions were due to transition errors. In this study, only one transition error was identified. However, our study did not assess readmissions. The explanation could be that not all transition errors have been revealed. Pharmacists could play a role in managing patient electronic medication records both in the hospital (medication reconciliation, discharge list) and in the pharmacy (over-the-counter medications) and potentially reduce the discrepancies in the medication history.

Howard et al. suggested concentrating interventions on the drug groups that accounted for more than half of the drug groups associated with preventable DRAs (antiplatelets, diuretics, NSAIDs, and anticoagulants). In our study, Anti-inflammatory and antirheumatic products, Antithrombotic agents, Drugs used in diabetes were the medication classes that accounted for more than half of the medication classes associated with preventable DRAs related to treatment safety. Diuretics, Antithrombotic agents, Drugs used in diabetes, and Agents acting on the renin-angiotensin system were the medication classes, which accounted for more than half of the medication classes associated with preventable DRAs related to treatment effectiveness.

Similarly, (Schmiedl et al., 2018), suggested regular individualized medication reviews of the most commonly implicated drugs in preventable DRAs. In this prospective multicenter, long-term study conducted in Germany (Schmiedl et al., 2018), the most frequently implicated drugs included digitoxin, low-dose acetylsalicylic acid, phenprocoumon, diclofenac, fast-acting insulin, glyburide (glibenclamide), spironolactone, torasemide, and intermediate-acting combined with fast-acting insulin. The most common preventability aspects included missing prevention strategies, relevant drug-drug interactions, and inappropriate drugs for age, body weight, and comorbidities.

In the prospective multicenter study from the Netherlands (Leendertse et al., 2008), medication classes associated most often with potentially preventable DRAs included antiplatelet drugs, oral anticoagulants, NSAIDs, and their combinations, antidiabetic drugs, and medications that act on the central nervous system. The most common medication errors associated with potentially preventable DRAs in the HARM study (Leendertse et al., 2008) included lack of a clear indication for the medication, nonadherence to the medication regimen, inadequate monitoring, and drug-drug interactions.

Epidemiological studies on preventable DRAs are constantly needed since clinical practice is changing as new preventive measures are being implemented. Compared to the past, lower target serum digoxin concentrations are recommended. Digoxin concentrations ≥1.2 ng/ml are avoided, since it has been shown to increase cardiovascular mortality (Rathore et al., 2003) and other ADEs. Lower doses of spironolactone are used in practice, and potassium levels and renal function are monitored following the publication that identified increased hyperkalemia-associated morbidity and mortality among patients treated with angiotensin-converting enzyme inhibitors and spironolactone (Juurlink et al., 2004). In the geriatric population, the goal is not too tight glycemic control, and sulfonylureas (especially glibenclamide) are prescribed less often.

Academicians should assess potential options that exceed the obligatory demands. Additional efforts are still needed to identify evidence-based interventions during sick days. Recently, the absence of a sick day management plan was identified to be among the root causes of preventable ADEs (de Lemos et al., 2021). Similarly, in our study, DRAs were related to acute illness accompanied by dehydration. However, randomized controlled trials that access the risks and benefits of temporarily stopping angiotensin-converting enzyme inhibitors/angiotensin II receptor blockers are still needed.

In addition, there is a need for the development of safe and effective medications for chronic pain. On the one hand, NSAIDs contribute to DRAs related to the gastrointestinal tract. On the other hand, opioids pose a risk of opioid dependence and addiction and other ADEs.

In the same way, the preventability aspects of DRAs related to treatment effectiveness will also change over time. There is still a huge burden of diseases affecting the cardiovascular system on hospital admissions. Recently, SGLT2 inhibitors (empagliflozin or dapagliflozin) have been recommended in certain patients with heart failure. Underuse of these medications could become a new DRP that contributes to hospital admissions of patients with heart failure with reduced ejection fraction. In addition, target low-density lipoprotein cholesterol levels for cardiovascular disease prevention have been modified. Last but not least, addressing medication nonadherence might get a greater awareness in the future.

### Interpretation

Recently, it was suggested that the widespread use of a signal detection cut-off in descriptive prevalence studies may have contributed to the perception that harmful drug treatment is the major problem of health care (Wallerstedt et al., 2021). Therefore, it should be underlined that medications often pose a risk in certain situations and many ADEs are multifactorial in nature. The underlying causes are also related to the behavior of the patients (medication nonadherence and inappropriate lifestyle measures).

Wallerstedt et al. have another excellent point in stating that studies on DRAs in which the benefits of treatment are not captured may bring about the risk of unjustly discrediting pharmacotherapy. This view is supported by our finding that Antithrombotic agents and Diuretics were the common cause of DRAs related to treatment safety and simultaneously the most common cause of DRAs related to treatment effectiveness. Had we only included DRAs related to treatment safety, a layman not taking the benefit-risk balance into account could assume that these medications are rather harmful. On the one hand, the use of Antithrombotic agents was associated with bleeding events, but on the other hand, their underuse was associated with cases of thromboembolic stroke due to atrial fibrillation. Similarly, on the one hand, Diuretics were involved in electrolyte imbalances and prerenal failure. On the other hand, withdrawal of Diuretics was associated with decompensation of heart failure.

Wallerstedt et al. point out that an adverse event can be the consequence of a prudent benefit-risk evaluation and correct drug treatment. These observations are confirmed by our finding that only a minority of DRAs related to treatment safety were preventable. We agree with Wallerstedt’s statement that medication error would probably be the primary interest from a health care perspective as these events could possibly be prevented. However, we think that the information on non-preventable ADRs might also be valuable as it could prompt pharmaceutical companies to invest in the development of safer alternatives.

Wallerstedt et al. have also emphasized that problems that may just as well have been caused by the disease may be less relevant when quantifying a health care problem for health care decision making and suggested restricting the reported events to those with at least a probable causal relationship with drug treatment. Therefore, it should be emphasized that the prevalence of DRAs identified in this study (15.6%) included events with possible causality, contributory reasons of admission, and ADRs, which were not preventable as well. Our definition of DRA covered all manifest DRPs that were the main reason or contributed to hospital admission. If we took into account only manifest DRPs that were the main reason for hospital admissions, the prevalence of DRAs would be 7%. If we took only manifest DRPs with a certain or probable causal relationship into account, the prevalence of DRAs would be 6%.

### Strengths

The first strength of the study is that electronic medical records were used as a data source for DRA identification. It has been noted that spontaneous reporting or database methods of data collection underreport ADEs and ADRs compared to medical chart screening (Leendertse et al., 2010). Another advantage of using medical records is the possibility to detect some cases of DRAs related to treatment effectiveness. Electronic medical records capture important health information (e.g., presenting complaint, laboratory data, documented ADRs, previous falls, smoking status, smoking history, alcohol consumption) compared to administrative claims databases.

The second strength of the study is the method of DRA identification. Own definitions and assessments hinder the interpretation and comparison of different studies. This study followed a comprehensive guide, and both causality assessment and assessment of contribution to the hospital admissions were performed. We have not limited the identification of DRAs to the trigger list since trigger lists require constant updates whenever official guidelines are updated (Hedman, 2020). As described in the DRA adjudication guide (Thevelin et al., 2018), only manifest DRPs (DRPs that caused harm) that were the main reason or contributory reason for hospital admission were considered DRA. Drug-related laboratory deviations and ADEs that were present at admission but did not contribute to hospital admission were not included in the definition of DRA. However, they can be found in Supplementary Tables S4, S5.

The third strength is that the study assessed potential preventability and identified medication classes involved in potentially preventable DRAs as well as preventability aspects. As suggested by Wallerstedt et al., preventable DRAs should be the main concern of research, as DRAs, which can potentially be avoided, are of interest for clinical practice.

The fourth strength is the generalizability of the study. Most studies focus on specific departments. In this study, no exclusion criteria related to department were applied.

Additional strength could be the categorization of DRAs on DRAs related to treatment safety and DRAs related to treatment effectiveness. Although the latest guidelines focused on manifest DRPs, they have not suggested differentiating between problems and causes. Perhaps it could be useful to classify DRAs in a hierarchical manner, separate causes from problems, as was suggested for DRPs (van Mil et al., 2004).

### Limitations

The main limitation of this study is the retrospective data collection process. The gold standard method is a prospective evaluation of patient medical records, laboratory tests, and interviews with patients and care providers (Parameswaran Nair et al., 2018). The limitation related to retrospective data collection includes the absence of medication reconciliation, patient interview, medication adherence confirmation. Therefore, the finding that the prevalence of DRAs related to treatment effectiveness was not as high as the prevalence of DRAs related to treatment safety could be skewed since no patient interview was conducted, and medication nonadherence was only taken into account when explicitly stated in electronic medical records.

The second limitation is the inclusion of cases with a possible causal relationship. Recently, Wallerstedt et al. suggested restricting reported events to those with at least a probable causal relationship with drug treatment (Wallerstedt et al., 2021). Although this suggestion differs from the OPERAM DRA adjudication guide (Thevelin et al., 2018) and AT-HARM10 tool (Kempen et al., 2019), we have provided these results in Supplementary Tables S6–S10. The essential distinctions between probable causal relationship and possible causal relationship are that in the latter case, there may be another equally likely explanation for the event, and/or there is no information or uncertainty with regard to what has happened after stopping. Therefore, the case is classified as possible, not only when the event could also be explained by disease but also when the information on withdrawal is lacking. There are cases when a dechallenge cannot be performed (e.g., when the benefit of the medication is greater than the risks or patient death). However, with the inclusion of a possible causal relationship, there is a possibility of a non-drug-related explanation of the symptoms being classified as ADE. In our study, there were cases of hyperkalemia associated with a reduction in kidney function due to dehydration and events that were multifactorial (hyponatremia, fall, syncope). Coppes et al. (2021) have highlighted that the tools to identify DRAs have no scale to assess the medication-relatedness of hospital admission, so some cases might be identified as drug-related, but disease progression may play a larger role. Wallerstedt et al. indicated that medical doctors are more likely to attribute the hospital admission to exacerbation of disease while pharmacists tend to attribute the event to ADEs (Wallerstedt et al., 2021). Therefore, there is a possibility of over-attribution of conditions to ADEs. Several other issues arise in applying causality assessment algorithms to adverse drug events. There is a need to update the algorithmic methods to allow perfect applicability in all possible clinical scenarios accordingly or not with the terms of marketing authorization (Mascolo et al., 2017).

The third limitation is the heterogeneity of electronic medical records. Variability of the completeness of electronic medical records between departments might affect the results. In our study, the share of falls on DRAs might be underestimated as the electronic medical records from the department of surgery were insufficient to evaluate the causality of falls.

The last limitation is the assessment of inter-rater reliability. Fleiss cappa indicated slight agreement (0.09) between the raters. However, only the cases preselected by the main investigator have undergone consensus assessment, as the consensus assessment of each case would be time-consuming. However, given the fact that pharmacists tend to attribute adverse events rather to the medications than the disease, the risk of a potential miss will likely be small.

## Conclusion

The total prevalence of DRAs to University Hospital Hradec Králové *via* the emergency department was 15.6%. Of 195 DRAs, 74% DRAs were related to treatment safety, and 26% DRAs were related to treatment effectiveness. If we took only manifest DRPs that were the main reason for hospital admissions into account, the prevalence of DRAs would be 7%.

ADEs affecting Gastrointestinal disorders and Metabolism and nutrition disorders accounted for 38% of DRAs related to treatment safety. Cardiac disorders accounted for 32% of all DRAs related to treatment effectiveness.

DRAs related to treatment safety most frequently involved Antithrombotic agents, Antineoplastic agents, Diuretics, Corticosteroids for systemic use, and Beta blocking agents, while DRAs related to treatment effectiveness most frequently involved Diuretics, Antithrombotic agents, Drugs used in diabetes, Agents acting on the renin-angiotensin system, and Lipid modifying agents.

The potential preventability of DRAs was 51%. Anti-inflammatory and antirheumatic products, Antithrombotic agents, and Drugs used in diabetes represented were most frequently associated with preventable DRAs related to treatment safety. The medication classes with the highest of preventability included Anti-inflammatory and antirheumatic products, Psycholeptics, and Drugs used in diabetes. The most common preventable ADEs included gastroduodenal hemorrhage, hypoglycemia, and a depressed level of consciousness.

The preventability aspects involved in potentially preventable DRAs related to treatment safety included primarily problems with drug selection, inappropriate monitoring and problems with dose selection, and inappropriate lifestyle measures. On the contrary, medication nonadherence was the most common preventability aspect of potentially preventable DRAs related to treatment effectiveness.

## Data Availability

The raw data supporting the conclusion of this article will be made available by the authors, without undue reservation.
