# Supplementary material for: Drug-Related Hospital Admissions via the Department of Emergency Medicine: A Cross-Sectional Study From the Czech Republic
Source: Front Pharmacol. 2022 Jun 13;13:899151. doi: 10.3389/fphar.2022.899151 (PMC9236275; doi:10.3389/fphar.2022.899151)
Supplement: Supplementary file 1 [file DataSheet1.docx]

Supplementary Material

Supplement to: Očovská Z, Maříková M, Kočí J, Vlček J. Drug-related Hospital Admissions via the Department of Emergency Medicine: A Cross-sectional Study from the Czech Republic. *Front Pharmacol*. 2022; 13: 899151. doi: [10.3389/fphar.2022.899151](https://doi.org/10.3389/fphar.2022.899151)

This supplementary material has been provided by the authors to give readers additional information about their work.

Table of Contents

[1 Potentially preventable DRAs 2](#_Toc104685804)

[Supplementary Table 1: Drug-related problems involved in DRAs related to treatment safety 2](#_Toc104685805)

[Supplementary Table 2. Drug-related problems involved in DRAs related to treatment effectiveness 3](#_Toc104685806)

[2 Subgroup analysis 4](#_Toc104685807)

[Supplementary Table 3: Subgroup analysis 4](#_Toc104685808)

[3 Events that did not fulfill the definition of DRA 5](#_Toc104685809)

[Supplementary Table 4: List of drug-related laboratory deviations 5](#_Toc104685810)

[Supplementary Table 5: List of adverse drug events that were present at hospital admission 6](#_Toc104685811)

[4 Characteristics of DRAs with a probable causal relationship 7](#_Toc104685812)

[Supplementary Table 6: Medication classes involved in DRAs related to treatment safety with a probable causal relationship 7](#_Toc104685813)

[Supplementary Table 7: Medication classes involved in DRAs related to treatment effectiveness with a probable causal relationship 7](#_Toc104685814)

[Supplementary Table 8: Medication classes involved in potentially preventable DRAs related to treatment safety with a probable causal relationship 8](#_Toc104685815)

[Supplementary Table 9: Clinical manifestations of DRAs related to treatment safety with a probable causal relationship 9](#_Toc104685816)

[Supplementary Table 10: Clinical manifestations of potentially preventable DRAs related to treatment safety with a probable causal relationship and associated medications 10](#_Toc104685817)

# Potentially preventable DRAs

Supplementary Table 1: Drug-related problems involved in DRAs related to treatment safety

| Drug-related problems related to treatment safety | No. | Description |
| --- | --- | --- |
| Drug selection | 21 |  |
| - Inappropriate drug according to guidelines | 13 | nimesulide (for long-term treatment)  furosemide (for arterial hypertension)  doxazosin (safer alternative for benign prostatic hyperplasia exists)  nimesulide + ASA (history of GIT ulceration)  ibuprofen + warfarin (history of GIT ulceration)  amiodarone (history of syncope)  meloxicam + dabigatran (history of microcytic anemia)  bisoprolol (history of bradycardia)  dosulepin (inappropriate for older patients)  diclofenac (history of microcytic anemia)  diclofenac (history of microcytic anemia)  meloxicam (history of gastritis)  ibuprofen (history of GIT bleeding) |
| - No indication for the drug | 4 | low-dose acetylsalicylic acid (3), levodopa |
| - Inappropriate combination of drugs | 1 | haloperidol (+ morphine, fentanyl) |
| - No or incomplete drug treatment in spite of existing indication | 3 | omission of gastric acid suppressants despite prior gastritis or gastrointestinal ulcer:  naproxen, ibuprofen, ibuprofen (+ rivaroxaban) |
| Dose selection | 6 |  |
| - Drug dose too high | 5 | glimepiride (8 mg per day)  tramadol (450 mg per day)  diclofenac (100 mg in older patient)  tiapride (300 mg in renal impairment)  nadroparin (inappropriate dose per weight) |
| - Dosage regimen too frequent enough | 1 | metoprolol (100 mg four times a day) |
| Patient-related | 3 |  |
| - Patient takes more drugs than prescribed | 1 | tramadol + zolpidem (overdose) |
| - Patient unable to use the drug as directed | 2 | tiapride, insulin (patients with dementia) |
| Other | 20 |  |
| - No or inappropriate outcome monitoring | 11 |  |
| - - symptoms of bradycardia, heart rate |  | amiodarone (+ bisoprolol), verapamil, digoxin (+ nebivolol) |
| - - symptoms of bleeding, INR |  | warfarin (5) |
| - - blood glucose |  | insulin (2) |
| - - blood potassium |  | potassium chloride |
| - Inappropriate lifestyle measures | 9 |  |
| - - food intake |  | glimepiride, insulin |
| - - fluid intake |  | furosemide (2), digoxin, perindopril, amiloride (+ telmisartan) |
| - - smoking |  | hormonal contraceptives |
| - - heavy episodic alcohol consumption |  | warfarin |
| Total | 50 |  |

ASA: Acetylsalicylic acid (low-dose), GIT: Gastrointestinal, INR: International Normalized Ratio

Supplementary Table 2. Drug-related problems involved in DRAs related to treatment effectiveness

| Drug-related problems related to treatment effectiveness | No. | Medication classes involved | No. |
| --- | --- | --- | --- |
| Drug selection |  |  |  |
| - No or incomplete drug treatment in spite of existing indication | 7 | Antithrombotic agents | 2 |
|  |  | Antithrombotic agents + Lipid modifying agents | 2 |
|  |  | Agents acting on the renin-angiotensin system | 1 |
|  |  | Antianemic preparations | 1 |
|  |  | Thyroid therapy | 1 |
| Treatment duration |  |  |  |
| - Duration of treatment too short | 1 | Antithrombotic agents | 1 |
| Patient-related |  |  |  |
| - Patient takes less drug than prescribed or does not take the drug at all | 35 | Diuretics | 7 |
|  |  | Drugs used in diabetes | 6 |
|  |  | Agents acting on the renin-angiotensin system | 4 |
|  |  | Antibacterials for systemic use | 3 |
|  |  | Antithrombotic agents | 3 |
|  |  | Antianemic preparations | 2 |
|  |  | Immunosuppressants | 2 |
|  |  | Antithrombotic agents + Lipid modifying agents | 1 |
|  |  | Calcium channel blockers + Antithrombotic agents | 1 |
|  |  | Calcium channel blockers + Beta blocking agents + Diuretics  + Lipid modifying agents | 1 |
|  |  | Diuretics + Agents acting on the renin-angiotensin system  + Antithrombotic agents + Lipid modifying agents | 1 |
|  |  | Diuretics + Beta blocking agents | 1 |
|  |  | Intestinal antiinflammatory agents | 1 |
|  |  | Psychoanaleptics | 1 |
|  |  | Psycholeptics | 1 |
| Patient transfer related |  |  |  |
| - Medication reconciliation problem | 1 | Diuretics + Agents acting on the renin-angiotensin system | 1 |
| Other |  |  |  |
| - No or inappropriate outcome monitoring | 6 | Drugs used in diabetes | 2 |
|  |  | Diuretics | 2 |
|  |  | Antithrombotic agents | 1 |
|  |  | Diuretics + Beta blocking agents | 1 |
| Total | 50 |  |  |

DRA: Drug-related hospital admission

# Subgroup analysis

Supplementary Table 3 depicts how results change when looking at subgroups of patients.

Supplementary Table 3: Subgroup analysis

| Subgroups | All  Hospital admissions | DRAs | DRAs related to treatment  safety | DRAs related to treatment  effectiveness | Prevalence of DRAs | Preventable  DRAs | Preventable DRAs  related to treatment  safety |
| --- | --- | --- | --- | --- | --- | --- | --- |
| *Whole sample* | *1252* | *195* | *145* | *50* | *15.6%* | *100 (51%)* | *50 (34%)* |
| **Age** |  |  | | | | | |
| < 18 | 24 | 0 | - | - | - | - | - |
| 18-64 | 416 | 44 | 22 | 22 | 10.6% | 29 (66%) | 7 (32%) |
| ≥ 65 | 812 | 151 | 123 | 28 | 18.6% | 71 (47%) | 43 (35%) |
| **Sex** |  |  | | | | | |
| female | 570 | 91 | 72 | 19 | 16.0% | 46 (51%) | 27 (38%) |
| male | 682 | 104 | 73 | 31 | 15.2% | 54 (52%) | 23 (32%) |
| **Department** |  |  | | | | | |
| internal medicine | 610 | 134 | 101 | 33 | 22.0% | 68 (51%) | 35 (35%) |
| surgery | 320 | 18 | 14 | 4 | 5.6% | 10 (56%) | 6 (43%) |
| neurology | 126 | 13 | 6 | 7 | 10.3% | 10 (77%) | 3 (50%) |
| pulmology | 47 | 12 | 10 | 2 | 25.5% | 3 (25%) | 1 (10%) |
| oncology | 33 | 8 | 8 | 0 | 24.2% | 1 (13%) | 1 (13%) |
| anestesiology | 42 | 6 | 4 | 2 | 14.3% | 5 (83%) | 3 (75%) |
| psychiatry | 12 | 3 | 1 | 2 | 25.0% | 3 (100%) | 1 (100%) |
| dermatology | 2 | 1 | 1 | 0 | 50.0% | 0 | 0 |
| other | 60 | 0 | - | - | - | - | - |
| **Charlson Comorbidity Index** | | | | | | | |
| < 5 | 684 | 74 | 50 | 24 | 10.8% | 44 (59%) | 20 (40%) |
| ≥ 5 | 568 | 121 | 95 | 26 | 21.3% | 56 (46%) | 30 (32%) |

DRA: Drug-related hospital admission

# Events that did not fulfill the definition of DRA

Supplementary Table 4 lists Drug-related laboratory deviations without clinical manifestation (n=42).

Supplementary Table 4: List of drug-related laboratory deviations

| Medications involved | Value (upper limit) | No. |
| --- | --- | --- |
| Supratherapeutic INR | | 20 |
| - warfarin | INR 8.7 |  |
| - warfarin | INR 11 |  |
| - warfarin | INR 4 |  |
| - warfarin | INR 4.58 |  |
| - warfarin | INR 3.9 |  |
| - warfarin | INR 4.06 |  |
| - warfarin | INR 11.14 |  |
| - warfarin | INR 3.96 |  |
| - warfarin | INR 3.63 |  |
| - warfarin | INR 6.9 |  |
| - warfarin | INR 3.91 |  |
| - warfarin | INR 3.91 |  |
| - warfarin | INR 5.08 |  |
| - warfarin | INR 11.7 |  |
| - warfarin | INR 3.69 |  |
| - warfarin | INR 3.84 |  |
| - warfarin | INR 4.2 |  |
| - warfarin | INR 5.3 |  |
| - warfarin | INR 7.1 |  |
| - warfarin | INR 3.96 |  |
| Hyperkalemia | | 10 |
| - ramipril + irbesartan | K 5.2 mmol/L |  |
| - ramipril | K 5.6 mmol/L |  |
| - perindopril | K 5.9 mmol/L |  |
| - spironolactone + telmisartan | K 7.5 mmol/L |  |
| - spironolactone + perindopril | K 5.4 mmol/L |  |
| - spironolactone + amiloride + perindopril | K 9.0 mmol/L |  |
| - losartan | K 6.5 mmol/L |  |
| - telmisartan | K 5.7 mmol/L |  |
| - spironolactone | K 7.1 mmol/L |  |
| - ramipril | K 5.7 mmol/L |  |
| Hypokalemia | | 4 |
| - hydrochlorothiazide + indapamide | K 2.8 mmol/L |  |
| - hydrochlorothiazide | K 2.4 mmol/L |  |
| - metipamide | K 2.8 mmol/L |  |
| - furosemide + hydrochlorothiazide | K 2.9 mmol/L |  |
| Increased drug level |  | 4 |
| - valproic acid | 839.4 (693 µmol/L) |  |
| - levetiracetam | 285.3 (217 µmol/L) |  |
| - digoxin | 3.34 nmol/l (1.54 nmol/L) |  |
| - digoxin | 2.16 nmol/L (1.54 nmol/L) |  |
| Tachycardia | | 2 |
| - formoterol + fenoterol + ipratropium + tiotropium | 128 beats per minute |  |
| - fenoterol + vilanterol + umeclidinium + ipratropium + theophylline | 147 beats per minute |  |
| Hyponatremia | | 2 |
| - losartan | Na 127 mmol/L |  |
| - hydrochlorothiazide + amiloride | Na 119 mmol/L |  |

CK: Creatine kinase, INR: International Normalized Ratio

Supplementary Table 5 provides overview of adverse drug events that were present at admission, but did not contribute to hospital admissions (n=7).

Supplementary Table 5: List of adverse drug events that were present at hospital admission

| Sex | Age | Clinical Manifestation | Medications involved | Causality | Reason of hospital admission |
| --- | --- | --- | --- | --- | --- |
| male | 68 | Gastroduodenal hemorrhage | ASA + rivaroxaban | possible | Peripheral artery disease |
| female | 87 | Confusion | tramadol + zolpidem | possible | Microcytic anemia |
| male | 85 | Abnormal dreams | zolpidem + trazodone | probable | Decompensated heart failure |
| male | 81 | Somnolence | pregabalin | possible | Aspiration pneumonia |
| female | 70 | Constipation | olanzapine | possible | Myopericarditis |
| female | 71 | Nausea | theophylline | possible | Acute Kidney Injury |
| male | 79 | Somnolence | trazodone + quetiapine | possible | Clostridium difficile colitis |

ASA: Acetylsalicylic acid (low-dose)

In addition, there were six cases of drug therapeutic failure with no obvious cause associated with warfarin and five cases of intentional intoxications associated with medications acting on central nervous system.

# Characteristics of DRAs with a probable causal relationship

Supplementary Table 6: Medication classes involved in DRAs related to treatment safety with a probable causal relationship

| ATC group code | ATC group name | No. | % |
| --- | --- | --- | --- |
| B01 | Antithrombotic agents | 46 | 34.1 |
| L01 | Antineoplastic agents | 19 | 14.1 |
| M01 | Antiinflammatory and antirheumatic products | 11 | 8.1 |
| C03 | Diuretics | 9 | 6.7 |
| A10 | Drugs used in diabetes | 8 | 5.9 |
| N02 | Analgesics | 6 | 4.4 |
| C07 | Beta blocking agents | 5 | 3.7 |
| C09 | Agents acting on the renin-angiotensin system | 5 | 3.7 |
| N05 | Psycholeptics | 5 | 3.7 |
| J01 | Antibacterials for systemic use | 4 | 3.0 |
| H02 | Corticosteroids for systemic use | 3 | 1.5 |
| L04 | Immunosuppressants | 3 | 2.2 |
| C01 | Cardiac therapy | 2 | 2.2 |
| C08 | Calcium channel blockers | 2 | 1.5 |
| A12 | Mineral supplements | 1 | 0.7 |
| C02 | Antihypertensives | 1 | 0.7 |
| G03 | Sex hormones and modulators of the genital system | 1 | 0.7 |
| N03 | Antiepileptics | 1 | 0.7 |
| N04 | Anti-parkinson drugs | 1 | 0.7 |
| N06 | Psychoanaleptics | 1 | 0.7 |
| R03 | Drugs for obstructive airway diseases | 1 | 0.7 |
|  | Total | 135 | 100 |

DRA: Drug-related hospital admission, ATC: Anatomical Therapeutic Chemical

Supplementary Table 7: Medication classes involved in DRAs related to treatment effectiveness with a probable causal relationship

| ATC group code | ATC group name | | No. | % |
| --- | --- | --- | --- | --- |
| C03 | Diuretics | 7 | | 26.9 |
| A10 | Drugs used in diabetes | 5 | | 19.2 |
| J01 | Antibacterials for systemic use | 3 | | 11.5 |
| B01 | Antithrombotic agents | 2 | | 7.7 |
| C09 | Agents acting on the renin-angiotensin system | 2 | | 7.7 |
| C07 | Beta blocking agents | 2 | | 7.7 |
| C10 | Lipid modifying agents | 1 | | 3.8 |
| B03 | Antianemic preparations | 1 | | 3.8 |
| L04 | Immunosuppressants | 1 | | 3.8 |
| A07 | Intestinal antiinflammatory agents | 1 | | 3.8 |
| H03 | Thyroid therapy | 1 | | 3.8 |
|  | Total | | 26 | 100 |

DRA: Drug-related hospital admission, ATC: Anatomical Therapeutic Chemical

Supplementary Table 8: Medication classes involved in potentially preventable DRAs related to treatment safety with a probable causal relationship

| ATC group code | ATC group name | No. | % |
| --- | --- | --- | --- |
| M01 | Antiinflammatory and antirheumatic products | 11 | 28.9 |
| B01 | Antithrombotic agents | 8 | 21.1 |
| A10 | Drugs used in diabetes | 6 | 15.8 |
| N05 | Psycholeptics | 4 | 10.5 |
| C01 | Beta blocking agents | 2 | 5.3 |
| A12 | Analgesics | 1 | 2.6 |
| C03 | Anti-parkinson drugs | 1 | 2.6 |
| C07 | Cardiac therapy | 1 | 2.6 |
| G03 | Diuretics | 1 | 2.6 |
| N02 | Mineral supplements | 1 | 2.6 |
| N04 | Psychoanaleptics | 1 | 2.6 |
| N06 | Sex hormones and modulators of the genital system | 1 | 2.6 |
|  | Total | 38 | 100 |

DRA: Drug-related hospital admission, ATC: Anatomical Therapeutic Chemical

Supplementary Table 9: Clinical manifestations of DRAs related to treatment safety with a probable causal relationship

| MedDRA System Organ Class | No. | % | MedDRA Preferred Term | No. |
| --- | --- | --- | --- | --- |
| Gastrointestinal disorders | 21 | 26.6 | Gastroduodenal hemorrhage | 10 |
|  |  |  | Intestinal hemorrhage | 4 |
|  |  |  | Diarrhea | 2 |
|  |  |  | Gastric ulcer perforation | 2 |
|  |  |  | Esophagitis | 1 |
|  |  |  | Nausea | 1 |
|  |  |  | Abdominal discomfort | 1 |
| Metabolism and nutrition disorders | 11 | 13.9 | Hypoglycemia | 6 |
|  |  |  | Hyperkalemia | 2 |
|  |  |  | Hyperglycemia | 1 |
|  |  |  | Calciphylaxis | 1 |
|  |  |  | Hyponatremia | 1 |
| Blood and lymphatic system disorders | 10 | 12.7 | Bone marrow toxicity | 8 |
|  |  |  | Microcytic anemia | 2 |
| Nervous system disorders | 9 | 11.4 | Cerebral hemorrhage | 4 |
|  |  |  | Depressed level of consciousness | 5 |
| Vascular disorders | 7 | 8.9 | Hypotension | 3 |
|  |  |  | Hematoma | 3 |
|  |  |  | Hemorrhage | 1 |
| Respiratory, thoracic, and mediastinal disorders | 6 | 7.6 | Hemoptysis | 2 |
|  |  |  | Pulmonary embolism | 1 |
|  |  |  | Pulmonary alveolar hemorrhage | 1 |
|  |  |  | Interstitial lung disease | 1 |
|  |  |  | Epistaxis | 1 |
| Immune system disorders | 4 | 5.1 | Drug hypersensitivity | 4 |
| Renal and urinary disorders | 4 | 5.1 | Hematuria | 4 |
| Cardiac disorders | 2 | 2.5 | Bradycardia | 1 |
|  |  |  | Cardiomyopathy | 1 |
| Psychiatric disorders | 2 | 2.5 | Confusional state | 1 |
|  |  |  | Disorientation | 1 |
| Infections and infestations | 2 | 2.5 | Infection susceptibility increased | 2 |
| General disorders and administration site conditions | 1 | 1.3 | Fatigue | 1 |
| Total | 79 | 100 |  |  |

DRA: Drug-related hospital admission, MedDRA: Medical Dictionary for Regulatory Activities

Supplementary Table 10: Clinical manifestations of potentially preventable DRAs related to treatment safety with a probable causal relationship and associated medications

| MedDRA System Organ Class category | No. | MedDRA Preferred Term | No. | Associated Medication |
| --- | --- | --- | --- | --- |
| Gastrointestinal disorders | 13 | Gastroduodenal hemorrhage | 6 | ibuprofen (2)  meloxicam  nimesulide  warfarin  nadroparin |
|  |  | Intestinal haemorrhage | 2 | nimesulide  meloxicam |
|  |  | Gastric ulcer perforation | 2 | ibuprofen  naproxen |
|  |  | Diarrhea | 1 | levodopa |
|  |  | Esophagitis | 1 | diclofenac |
|  |  | Nausea | 1 | digoxin |
| Metabolism and nutrition disorders | 8 | Hypoglycemia | 6 | glimepiride (2)  insulin human (2)  insulin glargine  insulin lispro |
|  |  | Hyperkalemia | 2 | amiloride  potassium chloride |
| Nervous system disorders | 6 | Depressed level of consciousness | 4 | haloperidol  tiapride  dosulepin  tramadol + zolpidem |
|  |  | Cerebral hemorrhage | 2 | warfarin  acetylsalicylic acid |
| Respiratory, thoracic and mediastinal disorders | 3 | Hemoptysis | 1 | warfarin |
|  |  | Pulmonary embolism | 1 | hormonal contraceptives |
|  |  | Pulmonary alveolar hemorrhage | 1 | warfarin |
| Blood and lymphatic system disorders | 2 | Microcytic anemia | 2 | ibuprofen  diclofenac |
| Vascular disorders | 2 | Hematoma | 2 | warfarin (2) |
| Psychiatric disorders | 1 | Disorientation | 1 | tiapride |
| Cardiac disorders | 1 | Bradycardia | 1 | bisoprolol |
| General disorders and administration site conditions | 1 | Fatigue | 1 | metoprolol |
| Total | 37 |  |  |  |

DRA: Drug-related hospital admission, MedDRA: Medical Dictionary for Regulatory Activities
